# Effect of Blue Light Filter Intraocular Lens (IOL) on Colour Vision

**DOI:** 10.7759/cureus.62963

**Published:** 2024-06-23

**Authors:** Ashraf M Nouman

**Affiliations:** 1 Ophthalmology, Khartoum Eye Teaching Hospital, Khartoum, SDN; 2 Ophthalmology, Sudan Medical Specialization Board, Khartoum, SDN

**Keywords:** cataract, intraocular lens (iol) implantation, sudan, colour blindness, acquired tritanomaly, bf iols, colour vision

## Abstract

Purpose

The objective of this study is to know the effect of blue light filter (BF) intraocular lenses (IOL) on colour vision of Sudanese patiens as BF IOLs are relatively new in Sudan and its effect on Sudanese patients is not yet studied.

Methods

This was a descriptive cross-sectional observational study. Data collection was done through interview and pretested structured questionnaire containing three sections: (i) personal data, (ii) past medical and ocular surgical history, and (iii) visual acuity (VA), the anterior and posterior segment exam result, and the D15 photopic color vision test result.

Results

In this study, 206 eyes of 103 patients were enrolled. Of them, 57 (55.3%) were females and 46 (44.7 %) were males. The mean age of the participants was 58.38 years±11.488 (SD). Furthermore, 81 patients (78.64%) achieved normal D15 color vision test results, while 22 patients (21.4%) had abnormal results. There was a significant effect (P=.00) on photopic color vision perception. Finally, the gender-wise performance showed an insignificant difference (P=.933) with 78.3% of the males having normal D15 color vision test and the females having slightly better results with 78.9 %.

Conclusions

The results suggested that implantation of BF IOLs has a significant effect on photopic color vision perception.

## Introduction

Color vision is the ability to perceive differences between light composed of different wavelengths independently of light intensity [1]. Color vision defect, also called color blindness, is the inability or decreased ability to see colors or perceive color differences under normal lighting conditions [2]. Color blindness can be anomalous trichromatic or dichromatic: protanopia (red deficient), deuteranopia (green deficient), and tritanopia (blue deficient)[2]. 

According to the World Health Organization (WHO) assessment, cataracts are responsible for 94 million of global blindness and it remains the leading cause of blindness, which can only be treated by surgical removal of the cataractous lens and intraocular lens (IOL) implantation [3]. There are many designs of IOLs including (i) Flexible IOLs, introduced into the eye via an injector and made of different materials like acrylic, silicone, and Collamer, (ii) Rigid IOLs, made entirely from polymethylmethacrylate (PMMA), (iii) Sharp/square-edged optics IOLs associated with a significantly lower rate of posterior capsular opacification (PCO) compared with round-edged optics; (iv) Aspheric optics, to counteract corneal spherical aberration, (v)Toric IOLs, which have an integral cylindrical refractive component to compensate for pre-existing corneal astigmatism, (vi) Multifocal IOLs, which provide clear vision over a range of focal distances, (vii) Adjustable IOLs, which allow the alteration of refractive power following implantation, (vii) Heparin coatings, which reduces the attraction of inflammatory cells and have particular application in eyes with uveitis, and (ix) Blue Light filters (BF), which include filters for blue wavelengths that impart a slight yellow tint to the IOL, similar to that of the physiological young adult lens [4].

Although essentially all IOLs contain filters for harmful ultraviolet light (UV) (200-400 nm) [4], the blue wavelengths of the visible spectrum (400-450 nm) can also cause damage to the retinal pigment epithelium (RBE) [5], and might exacerbate age-related macular degeneration (AMD) [6]. Hence, there was an impetus to develop BF IOLs, which proved to have many advantages such as decreasing photophobia and cynopsia [7] and glare [8], reducing optical chromatic aberration, improving contrast sensitivity [9], preventing the development of AMD [6] and the damage to RPE [5], and reducing blood-retinal barrier disruption [10]. Some evidence suggests a slightly poorer visual function in scotopic conditions of illumination as a disadvantage [4]. But will BF IOLs affect color vision? This was the main question in this study as BF IOLs are relatively new in Sudan. However, the majority of international studies have shown that there is no significant effect [11]. Most of the IOLs used today in Sudan are colorless but the BF IOLs are slightly yellowish, which might cause a change in the nature of absorbed light and affect color vision subsequently.

All the studies that were found are slightly different from our study in that they compare the effect of BF IOL on contrast sensitivity, color perception, and macular function to the effect of UV light filter (clear) IOL. While we are going to compare the effect of BF IOL on color perception with the normal lens of the other eye, we will mention some of the former too.

In a study that was done at the University of Sao Paulo, Brazil, on the effects of BF IOLs on the macula, contrast sensitivity, and color vision after a long-term follow-up, 60 eyes of 30 patients who received a UV and BF IOL in one eye and an acrylic UV light-filtering only IOL in the fellow eye were included, and it was concluded that after five years, there were no significant differences in color perception, scotopic contrast sensitivity, or photopic contrast sensitivity between the BF IOL and the IOL with a UV-light filter only [12]. The potential advantage of the tinted IOL in providing protection to macular cells remains unclear.

Another study was done at the Medical University of Vienna, Austria, on an intra-individual comparison of color contrast sensitivity in patients with clear and BF IOLs [13]. A UV light-filtering IOL was implanted in one eye and BF IOL was implanted in the contralateral eye. The study concluded that in this intra-individual comparison, the implantation of BF IOL did not lead to a clinically significant change in color perception and contrast sensitivity [13].

In Hospital NISA Valencia al Mar, Spain, a study was done on the comparison of contrast sensitivity and color discrimination after clear and yellow IOL implantation. Forty eyes of 20 patients were enrolled; one eye was implanted with UV light-filtering IOL and BF IOL was implanted in the contralateral eye. After postoperative follow-ups, they concluded that BF IOL is more advisable because they are capable of protecting the retina against UV light without disturbance of contrast sensitivity and chromatic vision [14].

Ankara University School of Medicine, Turkey, conducted a study on the effect of a yellow IOL on scotopic vision, glare disability, and blue color perception. The right eyes of 38 patients with a BF IOL and the right eyes of 38 age-matched patients with a conventional UV light-filtering IOL were included in the study. The conclusion was BF IOL provided contrast sensitivity under photopic and scotopic conditions (with and without glare) and blue color perception was comparable to that obtained with conventional UV light-filtering IOL. Scotopic vision and blue color discrimination decreased with age with both IOLs [15].

In conclusion, all the studies that we have reviewed state that BF IOL has either negligible effect on color vision and perception or no effect at all.

The general objective of this study was to see the effect of BF IOL on color vision, while the specific objectives were to check the color vision of the operated eye in the follow-up visit, to check the color vision of the healthy eye, and to compare the results. 

## Materials and methods

This was a descriptive, cross-sectional observational study. It was conducted in the outpatient clinic of the Noor Aleioon Hospital. All patients with BF IOL who visited the outpatient clinic for their one-month postoperative follow-up, all patients who underwent unilateral cataract surgery with BF IOL implantation and with a normal other eye were included in this study whereas patients with a single eye or those with problems in the other phakic eye, patients with bilateral cataract extraction operation, patients with clear IOL implantation and patients with old problems affecting color vision such as glaucoma, significant chorioretinal atrophy, congenital color vision defects and systemic diseases, particularly diabetes, were excluded from the study.

The sample size was calculated according to Robert Masson's equation: N=Z2 PQ/D 2 where N = sample size, Z = 1.96 at 95% confidence interval, P = probability, Q = (1-p) or percentage of failure, D = sampling errors. Our sample size was calculated as 103. 

The data collection was done through interviews with patients using a pretested structured questionnaire. All the patients who were asked to participate in the study kindly agreed. The questionnaire contained three sections. The first included personal data. The second contained the past medical and ocular surgical history. The third part contained the visual acuity (VA), the anterior and posterior segment exam results, and the D15 photopic color vision test result. All patients had a clear corneal incision, phacoemulsification, in-the-bag BF IOL implantation, and normal anterior and posterior segment examination. 

The data was coded and entered into IBM SPSS Statistics for Windows, Version 20.0 (Released 2011; IBM Corp., Armonk, New York, United States). The analysis was done by the same program in association with Microsoft Excel 2007 (Microsoft Corporation, Redmond, Washington, United States).

## Results

A total of 206 eyes of 103 patients were enrolled in this study, out of whom 57 (55.3%) were females and 46 (44.7%) were males (Table 1). The mean age of the participants was 58.38 years ± 11.488 (SD).

**Table 1:** Gender-wise participation (N=103)

Gender	Frequency	Percentage
Male	46	44.7%
Female	57	55.3%

**Figure 1:**
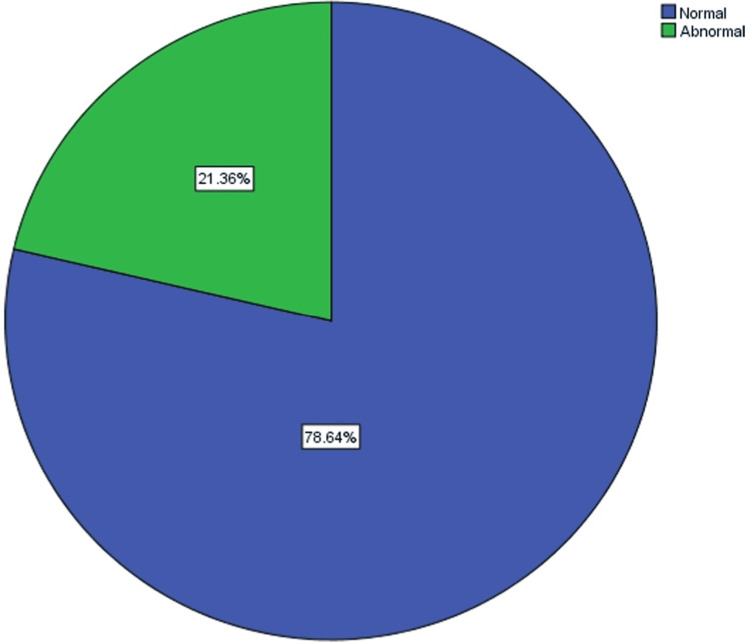
General performance of the operated eyes in D15 color vision test

Regarding the general performance of the participants' operated eyes in the D15 color vision test, 81 patients (78.64%) achieved normal test results, while 22 patients (21.4%) had abnormal results (Figure 1).

**Figure 2:**
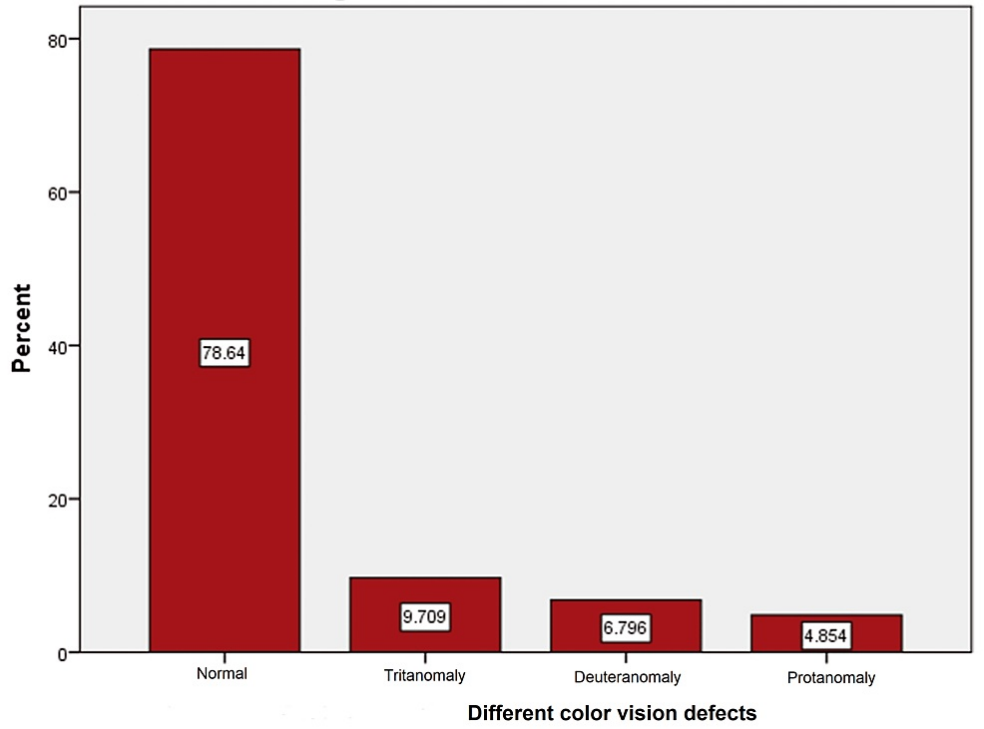
Percentage of different color vision defects in the operated eyes

Of the 22 patients (21.4%) with abnormal results, tritanomaly (blue deficient) constituted 9.709% (n=10), and deuteranomaly (green deficient) and protanomaly (red deficient) constituted 6.796% (n=7) and 4.854% (n=5), respectively (Figure 2).

Finally, the gender-wise performance showed an insignificant difference with 78.3% of the males having normal D15 color vision test results whereas the females had slightly better results with 78.9% having normal results (Table 2).

**Table 2:** Gender-wise performance in the D15 color vision test

General Performance	Gender	
	Male (N=46), n (%)	Female (N=57), n (%)
Normal Performance	36 (78.3%)	45 (78.9%)
Abnormal Performance	10 (21.7%)	12 (21.1%)

## Discussion

The effect of BF IOLs on color vision in Sudanese patients is not well studied. So, we conducted a study in which 206 eyes of 103 patients were enrolled. Regarding the general performance of the operated eyes in the D15 color vision test, 81 patients (78.64%) achieved normal test results, while 22 patients (21.4%) had abnormal results (Figure 1). In the present study, a significant effect (P=.00) was seen on photopic color vision perception compared to the negligible, insignificant effect shown by the majority of the international studies. This difference can be attributed to many factors: Firstly, the majority of the international studies are slightly different from our study in that they compare the effect of BF IOLs on contrast sensitivity, color perception, and macular function to the effect of UV-light filter (clear) IOLs, while we compare the effect of BF IOLs on color perception only with the normal lens of the other eye. Other causes might be the length of the study and the number of participants; our study was of a very short duration not exceeding six months, while the other studies took as much as five years. Furthermore, most of the international studies did not involve more than 40 participants as opposed to 103 in our study.

About 78.64% of the operated eyes had normal color vision while 21.36% had defective color vision and out of this defective percentage the tritanomaly (blue deficient) constituted 9.709% (P=.00). This defect is mainly attributed to the effect of the yellow tint of the BF IOLs, although there may be other factors. Deutranomaly (green deficient) and protanomaly (red deficient) constituted 6.796% and 4.854%, respectively; these defects were probably caused by lack of attention or undiscovered color vision defect since childhood.

Finally, the gender-wise performance showed insignificant differences (P=.933), which was against the author's initial expectation, with 78.3% of the males having normal D15 color vision test results whereas the females had slightly better results with 78.9% (Table 2).

Limitations

There were two main limitations. First, It was very difficult to find the D 15 color vision test here in Sudan. Also, the D 15 color vision test took a lot of time and concentration and was difficult for most of the patients, especially males and elderly patients. 

Recommendations

The needed equipment like the D 15 color vision test should be provided in the different ophthalmic institutes. More studies should be done about the effect of BF IOLs on color perception, contrast sensitivity, and macular protection to understand its feasibility on Sudanese patients. Finally, since this type of IOL will, to some degree, affect color perception, it should be avoided in patients employed in jobs that need intact color perception such as artists and pilots in order to not interfere with their work.

## Conclusions

This study was done to evaluate the effect of BF IOLs on the color vision of Sudanese patients as BF IOLs are relatively new in Sudan and their effect is not yet studied. A total of 206 eyes of 103 patients were enrolled in this study, and 22 patients (21.36%) had abnormal results (p=.00). This results suggest that implantation of BF IOLs has significant effect on photopic colour vision perception.

## Appendices

Questionnaire 

Section A

1. Name:       

2. Age:       

3. Gender:      Male   O                                Female   O

Section B

Past medical history:       

1. DM:              Yes    O                                 No   O       

2. Glaucoma:   Yes    O                                No   O       

3. Colour blindness: Yes   O                         No   O       

4. Other ocular diseases & surgeries: . . . . . . . . . . . . . . . . . . . . . .       

5. The operated eye:   RT   O                       Lt   O       

6. Period after the operation: . . . . . . . . . . . . . . . . . . . . . . . . . . . . .

Section C

1. Refraction:

The operated eye:

The other eye:                                                       

                                                                           RT                                         LT

2. Anterior segment exam:

3. Posterior segment exam:     

4. D15 color test report:

The operated eye:   

The other eye:  
